# Development of a prognostic model based on different disulfidptosis related genes typing for kidney renal clear cell carcinoma

**DOI:** 10.3389/fphar.2024.1343819

**Published:** 2024-03-13

**Authors:** Yuanyuan Feng, Wenkai Wang, Shasha Jiang, Yongming Liu, Yan Wang, Xiangyang Zhan, Huirong Zhu, Guoqing Du

**Affiliations:** ^1^ Department of Oncology, Shuguang Hospital Affiliated to Shanghai University of Traditional Chinese Medicine, Shanghai, China; ^2^ Shi’s Center of Orthopedics and Traumatology, Shuguang Hospital Affiliated to Shanghai University of Traditional Chinese Medicine, Shanghai, China; ^3^ Urology Centre, Shuguang Hospital Afliated to Shanghai University of Traditional Chinese Medicine, Shanghai, China

**Keywords:** kidney renal clear cell carcinoma (KIRC), disulfidptosis, prognostic model, immune cell infiltration, tumor microenvironment, risk signature

## Abstract

**Background:** Kidney renal clear cell carcinoma (KIRC) is a common and clinically significant subtype of kidney cancer. A potential therapeutic target in KIRC is disulfidptosis, a novel mode of cell death induced by disulfide stress. The aim of this study was to develop a prognostic model to explore the clinical significance of different disulfidptosis gene typings from KIRC.

**Methods:** A comprehensive analysis of the chromosomal localization, expression patterns, mutational landscape, copy number variations, and prognostic significance of 10 disulfide death genes was conducted. Patients were categorized into distinct subtypes using the Non-negative Matrix Factorization (NMF) typing method based on disulfidptosis gene expression patterns. Weighted Gene Co-expression Network Analysis (WGCNA) was used on the KIRC dataset to identify differentially expressed genes between subtype clusters. A risk signature was created using LASSO-Cox regression and validated by survival analysis. An interaction between risk score and immune cell infiltration, tumor microenvironment characteristics and pathway enrichment analysis were investigated.

**Results:** Initial findings highlight the differential expression of specific DRGs in KIRC, with genomic instability and somatic mutation analysis revealing key insights into their role in cancer progression. NMF clustering differentiates KIRC patients into subgroups with distinct survival outcomes and immune profiles, and hierarchical clustering identifies gene modules associated with key biological and clinical parameters, leading to the development of a risk stratification model (LRP8, RNASE2, CLIP4, HAS2, SLC22A11, and KCTD12) validated by survival analysis and predictive of immune infiltration and drug sensitivity. Pathway enrichment analysis further delineates the differential molecular pathways between high-risk and low-risk patients, offering potential targets for personalized treatment. Lastly, differential expression analysis of model genes between normal and KIRC cells provides insights into the molecular mechanisms underlying KIRC, highlighting potential biomarkers and therapeutic targets.

**Conclusion:** This study contributes to the understanding of KIRC and provides a potential prognostic model using disulfidptosis gene for personalized management in KIRC patients. The risk signature shows clinical applicability and sheds light on the biological mechanisms associated with disulfide-induced cell death.

## Introduction

Renal cell carcinoma (RCC) is the second-most common cause of death associated with malignant neoplasms of the urinary tract (Li et al., 2017). A series of possible features have been recognised, such as environmental factors (e.g., smoking, obesity, high blood pressure, long-term use of painkillers) and genetic factors, which are related to the renal cell carcinoma (Hsieh et al., 2017). KIRC is one type of RCC and constitutes 80% of all renal malignancies (Klümper et al., 2020; Chu et al., 2023). Although, the therapeutic efficacy of KIRC has improved dramatically due to rapid advances in medical treatment and research. However, the majority of patients with metastatic disease have a poor prognosis and limited therapeutic advances due to few or no early symptoms of KIRC (Zhuang et al., 2019). In this way, early detection of KIRC contributes to a better prognosis (Li et al., 2019). Understanding KIRC is of paramount importance for both clinical practice and research aimed at improving therapeutic outcomes for patients with this disease, and the identification of clear and reliable novel prognostic molecular biomarkers can accurately predict the prognosis of KIRC patients.

Disulfidptosis, a recently proposed mode of cell death, is a novel phenomenon with important implications for understanding and treating cancer. When cells are starved of glucose and solute carrier family 7 member 11 (SLC7A11) is expressed at high levels, it induces disulfidptosis and initiates the cell death process. The reduction-oxidation (REDOX) reaction and the formation of disulfide bonds are the mechanisms that trigger cell death (Wang et al., 2023). In a groundbreaking study by Liu et al., it was demonstrated that disulfidptosis is induced by disulfide stress and rapid cell death in SLC7A11 high-expression cells, specifically under conditions of glucose starvation (Liu et al., 2023). Abnormal accumulation of cystine in the cytoplasm leads to disulfide stress, forcing the reduction of cystine to the more soluble cysteine, which produces relatively strong cytotoxicity (Liu X. et al., 2021). The reducing of cystine to cysteine requires the consumption of reducing equivalents of nicotinamide adenine dinucleotide phosphate (NADPH), so SLC7A11-expressing cells with a high rate of cystine uptake demand a high level of NADPH to continuously reducing cystine to cysteine to support intracellular homeostasis. NADPH production is impaired by the intracellular accumulation of cystine and other disulfide molecules and rapid cell death in response to glucose starvation, known as disulfidemia (disulfidptosis) (Liu et al., 2020; Yan et al., 2023). Cancer development and progression are closely linked to the formation and cleavage of disulfide bonds. Sulfur (S)-based chemical bonds have been used to develop tumour-specific, redox-responsive DDSs, which include the thioether bond, the disulfide bond and the thioketal bond (Sun et al., 2019). Notably, this unique pattern of cell death was identified in a kidney cancer cell line, suggesting its relevance and possible potential as a treatment target in the context of KIRC (Zhang et al., 2022; Kang et al., 2023). In spite of these ground-breaking findings, the full impact of disulfide on the pathogenesis, progression, and treatment of KIRC remains to a large extent unexplored.

The study was designed to determine the clinical significance and predictive potential of the risk profile based on the expression pattern of the disulfide disulfidptosis genes in patients with KIRC. To gain insight into potential mechanisms of disulfide-induced KIRC cell death, the study also aimed to investigate the association of risk scores with immune cell infiltration, tumor microenvironment characteristics, drug sensitivity, and pathway enrichment analyses.

## Methods

### TCGA data acquisition and clinical details

RNA-seq transcriptome data and relevant clinical details, including sex, age, subtype, IDH status, and survival information for KIRC, were taken from the Cancer Genome Atlas (TCGA)-KIRC database, available at https://portal.gdc.cancer.gov/. In addition, somatic mutation counts and copy number variation (CNV) data were also obtained from the TCGA repository.

### Comprehensive analysis of disulfidptosis-related genes (DRGs)

This study first comprehensively examines the chromosomal localization, differential expression patterns, mutational landscape, copy number variations, and prognostic significance of 10 disulfide death genes in KIRC cells. And the 10 disulfidptosis-associated genes (GYS1, NDUFS1, OXSM, LRPPRC, NDUFA11, NUBPL, NCKAP1, RPN1, SLC3A2, and SLC7A11) was obtained from previous literature (Liu et al., 2023). To accomplish this, the genomic positions of the 10 disulfide death genes on chromosomes were precisely mapped. The chromosome and gene positions were obtained from the NCBI database (https://www.ncbi.nlm.nih.gov/). And the changes in gene expression levels were compared between KIRC cells and healthy controls. Moreover, the mutational profiles and copy number variations of these genes specifically within KIRC cells were investigated. Lastly, the potential prognostic value of the ten genes in KIRC was explored. These data are all from the TCGA-KIRC project and were visualized using ggplot2 R package (version 3.4.4).

### NMF typing of clear cell carcinoma patients based on ten disulfide death genes

KIRC patients were classified into distinct subtypes using the NMF typing method, which uses a panel of 10 disulfide death genes. The NMF clustering approach, implemented by the NMF R package (version 0.26), was used for this purpose. The study applied specific criteria to determine the optimal configuration, including the evaluation of the consistency map, the comprehensive coefficient and the silhouette coefficient. In particular, the analysis included ten runs with the number of clusters (k) set to 2. The resulting patient subtypes were defined according to the distribution of 10 disulfide death genes. Kaplan-Meier analysis was then performed to generate survival curves (Xu et al., 2022) by the survival R package (version 3.5–7). The log-rank test was applied to compare disease-specific survival differences between the two risk groups and to assess their significance by the stats R package (Nomura et al., 2020). The differences in immune cell infiltration, tumour microenvironment and stemness index between the two clusters were evaluated by the estimate R package (version 1.0.13).

### Differential expression and WGCNA analysis of DEGs between clusters

Differential expression analysis of genes (DEGs) between cluster C1 and cluster C2 was performed using the limma R package (version 3.50.3). This study utilized WGCNA to buid a gene co-expression network and divide genes into five modules, and studied the gene significance and the relationship between the module and phenotype by using the WGCNA R package (version1.72–5) (Gao et al., 2022).

### Development and validation of a risk signature for survival prediction in KIRC

Based on univariate Cox analysis, we identified survival-associated genes from the most important module and established a risk signature consisting of six genes (LRP8, RNASE2, CLIP4, HAS2, SLC22A11, and KCTD12) using LASSO-Cox regression with the glmnet R package (version 4.1–8) and survival R package (version 3.5–7). After constructing the risk score, we employed Cox regression models, including univariate and multivariate Cox proportional hazards regression models, to investigate the effect of various factors, including the risk score, on survival time (Xue et al., 2022). The TCGA samples were divided into a training set and a test set to validate our model, and with reference to the median score, we classified the KIRC patients into a low-risk group and a high-risk group. The Kaplan-Meier survival curve was then used to compare the overall survival (OS) and progression-free survival (PFS) of the two groups of patients (Ohashi et al., 2020). The concordance index, which represents the proportion of concordant pairs between predicted and observed outcomes across all patients, was used to evaluate the predictive ability of the model. The time-dependent ROC curve (timeROC) R package (version 0.4) was employed to calculate time-dependent receiver operating characteristic (ROC) curves for sensitivity and specificity based on the signature (Park et al., 2022). Using the rms R package (version 6.7-1), we constructed calibration plots, calibration curve plots, and nomograms for 1-year, 3-year, and 5-year survival based on the risk score and other significant clinical features. These evaluations were conducted to assess the clinical applicability of the risk model.

### Estimation of immune and stromal cell abundance in the tumor microenvironment

The Microenvironment Cell Populations-counter (MCP counter) was used to estimate the absolute abundance of eight immune cell types in the tumour microenvironment, including T cells, CD8 T cells, cytotoxic lymphocytes, B lineage, NK cells, monocytic lineage, myeloid dendritic cells, and neutrophils, and two stromal cell types (endothelial cells and fibroblasts) (Horr and Buechler, 2021). CIBERSORT-ABS was employed to calculate the abundance of immune-related cells (Feng et al., 2022). The relationship between risk levels of immune cell infiltration and clusters was investigated using the limma R package. Furthermore, the linkET package was used to estimate the association between risk scores, immune checkpoint-related genes, immune cell infiltration, and immune function using Spearman’s correlation analysis (Wang et al., 2022). Additionally, this study compared the tumor mutation burden and mutation status of genes between high-risk and low-risk groups. The impact of tumor mutation burden on prognosis was explored through Kaplan-Meier (KM) analysis (Zhao et al., 2021).

### Investigation of immunophenoscores and drug sensitivity

The Immunophenoscores (IPS) of KIRC patients were collected from the Cancer Immunome Atlas (TCIA, https://tcia.at/home) database, and the correlation of IPS with risk features was investigated using the Wilcoxon rank-sum test (Liu Y. et al., 2021). The oncoPredict R software package (version 0.2) was employed to predict drug susceptibility scores for KIRC. The correlation between risk scores and drug susceptibility scores was then assessed using the limma R package and plotted using the ggplot2 R package to explore the clinical value of this risk model in the management of KIRC.

### Gene function and pathway analysis of disulfide-induced cell death

To elucidate the gene functions and biological pathways associated with disulfide-induced cell death, differential expression analysis of annotated genes was conducted using the R packages limma and clusterProfiler (version 4.2.2). Gene Ontology (GO) and Kyoto Encyclopedia of Genes and Genomes (KEGG) pathway enrichment analyses were performed on the identified differentially expressed genes. Subsequently, gene set enrichment analysis (GSEA) was conducted using the GSEA software (http://www.broadinstitute.org/gsea/index.jsp) on the gene sets from MSigDB (c2. cp.kegg.v7.4. symbols.gmt) version 4.0 (Jia et al., 2023). Furthermore, to investigate the biological functional differences among the groups, Gene Set Variation Analysis (GSVA) enrichment analysis was performed using the GSVA R package (Tang et al., 2023).

### Cell culture

786-O cells are derived from tissue of primary clear cell carcinoma, while CCC-HEK-1 cells are derived from normal human embryonic kidney tissue. 786-O cells were grown in complete medium (RPMI-1640 containing 10% fetal bovine serum (FBS) and 100 U mL^−1^ penicillin-streptomycin) in humidified air with CO_2_ at 37°C for 24 h. CCC-HEK-1 cells were grown in complete medium (DMEM containing 10% fetal bovine serum (FBS) and 100 U mL^−1^ penicillin-streptomycin) in humidified air with CO_2_ at 37°C for 24 h.

### Patients’ samples collection

The normal kidney and KIRC samples from patients were obtained from the Shuguang Hospital Affiliated to Shanghai University of Traditional Chinese Medicine, China. The normal kidney tissue samples were obtained from healthy controls (sample size = 3), and KIRC tissue samples were obtained from patients diagnosed with KIRC (sample size = 5). The Ethics Committee of the Shuguang Hospital Affiliated to Shanghai University of Traditional Chinese Medicine approved the protocol for collecting human samples (approval number 2023-1311-78-01).

### Quantitative real-time PCR

Total mRNA was extracted from cells and clinic samples using RNA Isolation Kit according to the manufacturer’s instructions. Then cDNA was synthesised using the First Strand cDNA Synthesis Kit according to the manufacturer’s instructions, which was treated as template DNA for the qRT-PCR assay. The qRT-PCR of six module genes (LRP8, RNASE2, CLIP4, HAS2, SLC22A11, and KCTD12) was performed using SYBR Green qPCR Mix on a real-time detector. Each experiment was repeated three times to calculate the mean and standard deviation (SD). The details of reagents and primers for the genes are in Supplementary Table S1.

### Data statistical analysis

All data in this study are expressed as mean standard deviation (SD). All bioinformatics analysis is performed using R software (v.4.2.2). *p* values were calculated using SPSS Statistics 25 (*p < 0.05, **p < 0.01, and ***p < 0.001).

## Results

### Identification and expression analysis of DRGs

**FIGURE 1:**
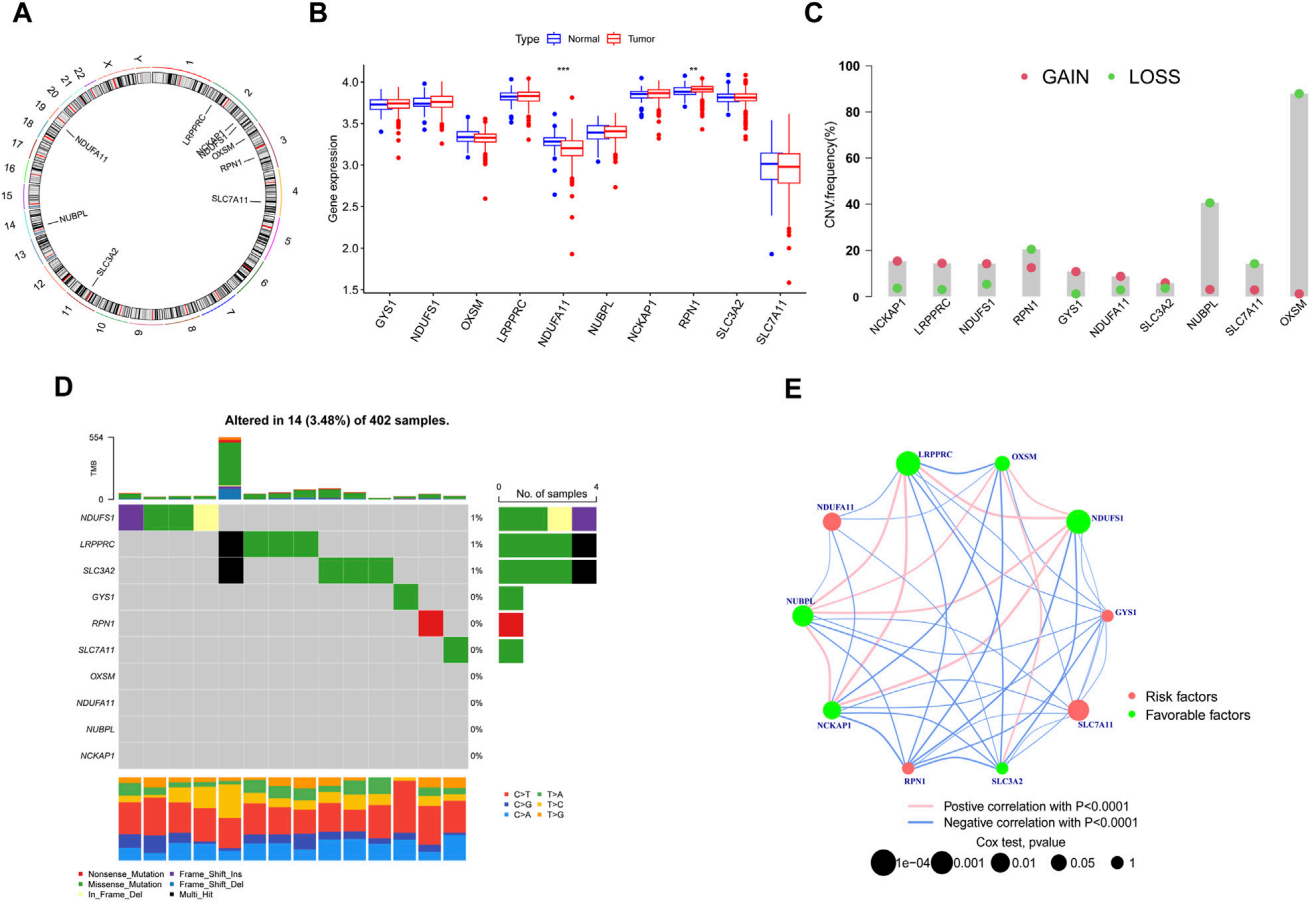
Identification and Expression Analysis of DRGs. **(A)** Chromosomal mapping. **(B)** Expression profiling. **(C)** CNV-related mutations. **(D)** Somatic mutation landscape. **(E)** Network Visual Analytics. (*p < 0.05, **p < 0.01, and ***p < 0.001).

In this investigation, we meticulously analyzed a cohort of 10 DRGs to elucidate their role in KIRC. Chromosomal mapping, as depicted in Figure 1A, delineates the precise genomic locations of these genes. Notably, expression profiling reveals a distinct downregulation of NDUFA11 in tumor tissues, in contrast to the upregulation observed for RPN1; the expression of the other genes remained statistically unaltered between normal and tumor samples (Figure 1B). Our study further extends into the realm of genomic instability, where Figure 1C highlights the prevalence of CNV-related mutations across these DRGs. In this context, OXSM is notably predisposed to frequent CNV deletions, whereas the remaining genes exhibit a spectrum of CNV deletions and amplifications. Additionally, Figure 1D provides an insightful overview of the somatic mutation landscape in these genes within KIRC, illustrating a uniformly low mutation rate. Complementing these findings, Figure 1E uses network visualisation techniques to intricately map the connections between DRGs and their prognostic significance in KIRC, providing a comprehensive and multifaceted view of their biological relevance.

### NMF clustering and tumor microenvironment analysis

**FIGURE 2:**
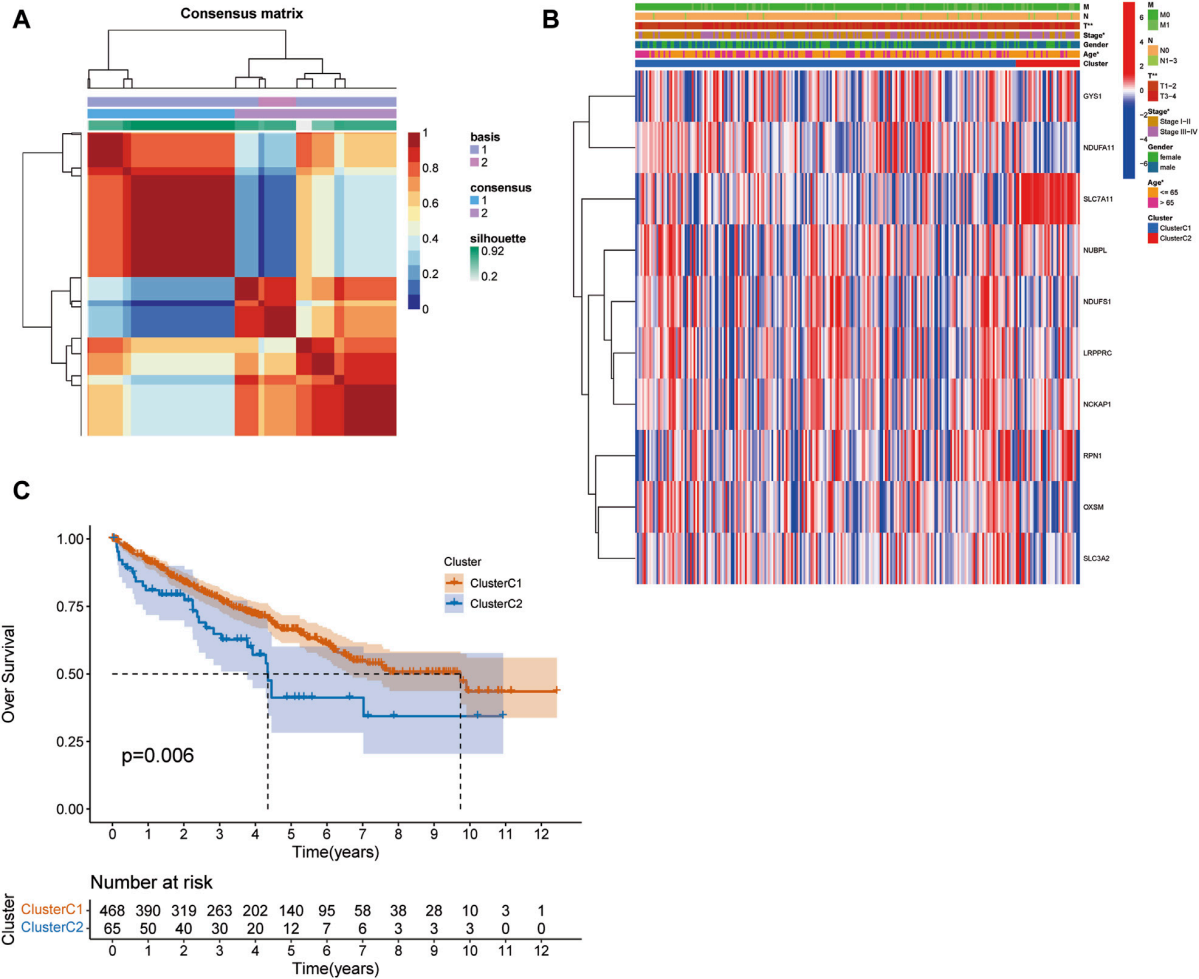
NMF Clustering and survival analysis. **(A)** The NMF algorithm clusters patients into two subgroups. **(B)** The expression patterns associated with DRGs in the C1 and C2 clusters. **(C)** Difference in survival between patients in clusters C1 and C2. (*p < 0.05, **p < 0.01, and ***p < 0.001).

We employed the NMF algorithm to stratify KIRC patients into two distinct subgroups, designated as C1 and C2. Our analysis elucidated a robust consistency matrix for these patient clusters (Figure 2A). Additionally, we profiled the differential expression patterns of DRGs within these subgroups, as visualized in a comprehensive heat map (Figure 2B). Crucially, Kaplan-Meier survival analysis (Figure 2C) underscored a statistically significant disparity in patient survival rates between clusters C1 and C2 (*p* = 0.009), indicating potential prognostic implications. In terms of tumor microenvironment (TME) characterization, the C1 cluster demonstrated a marked enrichment of specific immune cell infiltrates, including CD8 T cells, monocytes, and neutrophils (Supplementary Figure S1A), suggesting a unique immunological landscape. Further, TME and stemness indices were analyzed in the C1 and C2 cohorts (Supplementary Figure S1B). To better understand the impact of clusters on immune cell infiltration in KIRC, we examined the differences in the tumor microenvironment between the C1 and C2 subtypes (Supplementary Figure S1C). These findings provide critical insights into the heterogeneity of the tumor microenvironment and its potential impact on the clinical trajectory of KIRC patients, as detailed in Supplementary Material S2.

### Hierarchical clustering of genes and module analysis

**FIGURE 3:**
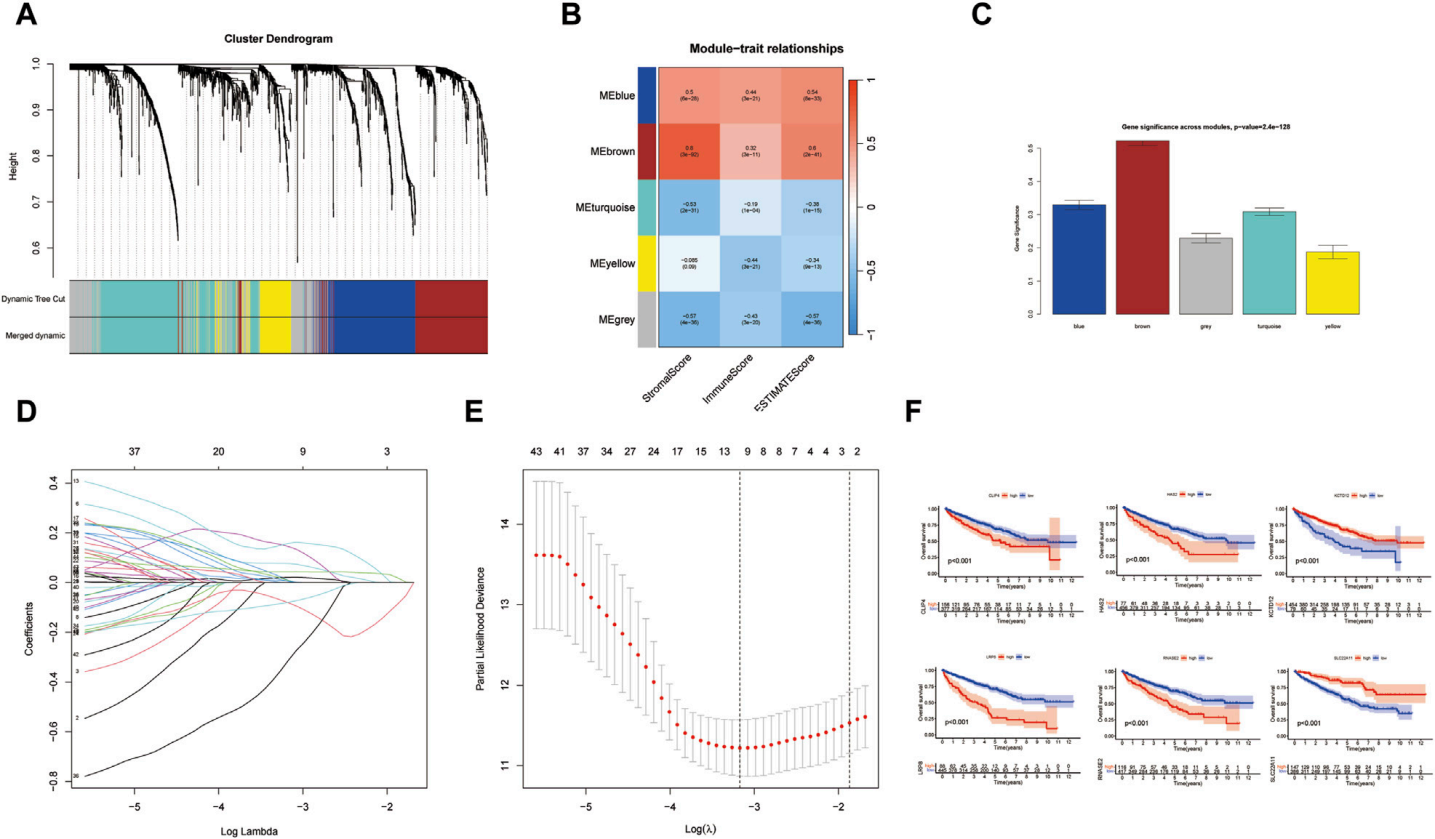
Hierarchical clustering methods for dividing genes into modules. **(A)** The cluster dendrogram. **(B)** Module-trait relationships. **(C)** Gene significance across modules. **(D,E)** Lasso model for screening model genes. **(F)** The KM survival analysis results for individual genes. (*p < 0.05, **p < 0.01, and ***p < 0.001).

**FIGURE 4:**
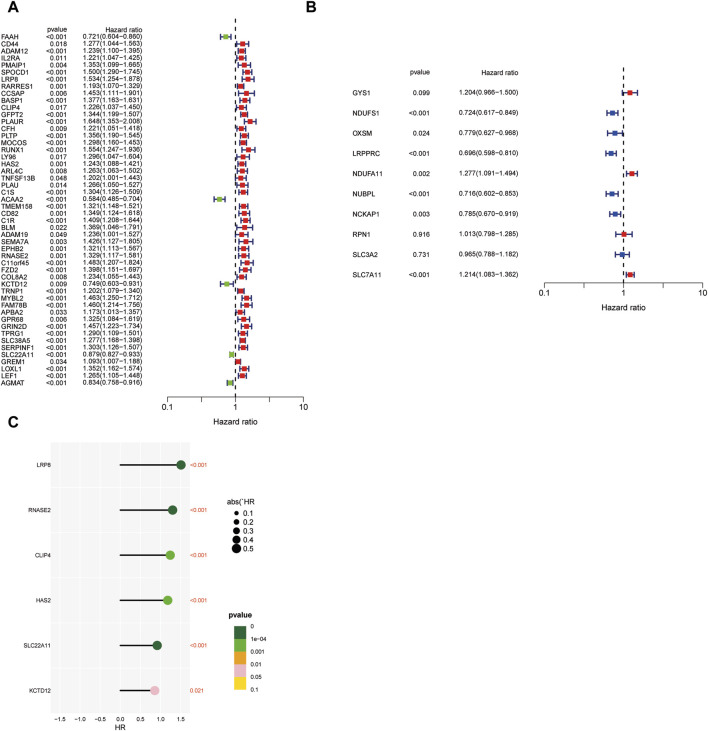
Predictive power of modeled genes. **(A)** Univariate Cox analysis of genes within each model. **(B)** Univariate Cox analysis of model genes. **(C)** The corresponding hazard ratio values and their associated *p*-values. (*p < 0.05, **p < 0.01, and ***p < 0.001).

Our study adopts a hierarchical clustering approach to partition genes into distinct modules, and the resulting cluster dendrogram is illustrated in Figure 3A. To elucidate the relationship between these modules and key biological parameters, Figure 3B provides a graphical representation of the associations between the five identified modules and Stromal Score, Immune Score, as well as ESTIMATE Score. It can be found that MEblue and MEbrown modules are negatively correlated with Stromal Score, Immune Score, and ESTIMATE Score, while the other three modules are positively correlated with Stromal Score, Immune Score, as well as ESTIMATE Score. Among them, MEbrown has the closest relationship with Stromal Score. Furthermore, Figure 3C presents a comprehensive assessment of the linkages between individual modules and various phenotypic traits, shedding light on their potential functional relevance. It was found that the relationship between MEbrown module and phenotypic traits is the most significant, followed by the MEblue module as the second most significant. To gain further insights into the prognostic significance of the identified gene modules, univariate Cox analysis was conducted for genes within each module, and the results are visually represented in Figure 4A Subsequently, a risk scoring system was constructed through the application of LASSO-Cox regression, wherein six pivotal genes were selected from the most crucial module, as elucidated in Figures 3D, E. In order to provide a more in-depth understanding of the predictive capabilities of the model genes, Figure 4B showcases the outcomes of univariate Cox analysis, while Figure 4C elucidates the corresponding hazard ratio (HR) values and their associated *p*-values, thus offering valuable insights into the potential prognostic significance of these genes. Additionally, Figure 3F depicts the KM survival analysis results for individual genes, further illuminating their impact on patient outcomes. These analytical findings collectively contribute to a comprehensive assessment of the molecular landscape under investigation, offering critical insights into the potential clinical relevance of the identified gene modules and their constituent genes.

### Risk stratification and validation of the risk model

**FIGURE 5:**
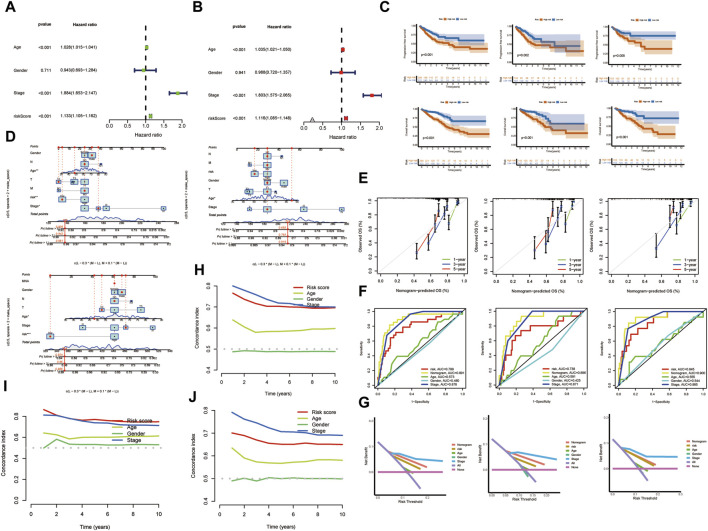
Risk Stratification and Validation of the Risk Model. **(A)** Univariate Cox regression analysis. **(B)** Multivariate Cox regression analysis. **(C)** Kaplan-Meier analysis. **(D)** The calibration plot. **(E)** Survival curves. **(F)** ROC curves. **(G)** Decision curves. **(H)** C-index. **(I,J)** Training and validation sets. (*p < 0.05, **p < 0.01, and ***p < 0.001).

Patients were categorized into low-risk and high-risk groups based on median risk scores. This stratification was pivotal in assessing the prognostic impact of the risk score. Figure 5A elucidates the outcomes of our univariate Cox regression analysis. It reveals a hazard ratio (HR) of 1.077 for the risk score [95% Confidence Interval (CI): 1.048–1.107, *p* < 0.001], signifying its significant prognostic value in the context of KIRC. Further, multivariate Cox regression analysis, detailed in Figure 5B, demonstrates an HR of 1.086 (95% CI: 1.050–1.122, *p* < 0.001) for the risk score. This analysis, accounting for various covariates, reinforces the risk score’s robustness as an independent prognostic indicator. To validate the prognostic model, TCGA samples were segregated into training and testing sets. Kaplan-Meier analysis, presented in Figure 5C, indicates a direct correlation between elevated risk scores and decreased overall survival (OS) and progression-free survival (PFS) in both sets, highlighting the predictive accuracy of our risk stratification approach. The calibration plot (Figure 5D) further underscores the clinical applicability of the risk score. It displays a remarkable concordance between predicted and observed survival outcomes, as evidenced by the calibration curves for 1-year, 3-year, and 5-year survival (Figure 5E). These curves exhibit a striking consistency between estimated and actual OS rates. ROC curves (Figure 5F) and decision curves (Figure 5G) further corroborate the risk score’s reliability. The ROC values (ROC train 0.845, test 0.738, all 0.788) demonstrate the model’s robust predictive capability. Lastly, the overall concordance index (c-index), depicted in Figure 5H, along with the c-index for the training (Figure 5I) and validation (Figure 5J) sets, confirms the model’s high predictive accuracy. These indices have reached satisfactory levels, reinforcing the model’s efficacy in forecasting clinical outcomes in KIRC patients. This comprehensive analysis solidifies the risk score as a vital tool in the prognostication of KIRC.

### Tumor microenvironment and immune infiltration analysis

We analyzed the TME and genetic profiles of patients with KIRC stratified into high-risk and low-risk groups based on their prognostic risk scores. Supplementary Figure S2A-C highlight that the high-risk group is characterized by a higher TME score, suggesting a more favorable prognosis for immunotherapy responsiveness. This observation underscores the potential for personalized therapeutic strategies based on risk stratification. In terms of the stemness index, the low-risk group exhibits an EREG-mRNAsi value closer to 1, indicative of a higher resemblance of tumor cells to stem cells. This finding may have significant implications for understanding the biology of lower-risk KIRC tumors. Supplementary Figure S2D further elucidates a strong association between the high-risk group and six types of immune infiltrating cells. This correlation may provide insights into the immunological underpinnings of high-risk KIRC. Tumor mutation burden (TMB) analysis shown no significant difference between high-risk and low-risk groups. However, Supplementary Figures S3B, C offer an in-depth view of the tumor gene mutation profiles for these groups, while Supplementary Figure S3D provides a comprehensive summary of the mutations, including variant classification and the top 10 mutated genes. Kaplan-Meier analysis (Supplementary Figure S3E) demonstrates that patients in the high H-TMB group exhibit a lower survival rate compared to those in the low H-TMB group (*p* < 0.001). Similarly, Supplementary Figure S3F shows that within the high-risk category, patients with high TMB have a lower survival rate than those in the low-risk group with low TMB (*p* < 0.01). Additionally, the differential expression of immune checkpoint-related genes is shown in Supplementary Figure S3J, with notable upregulation of TNF and HAVCR2 in the high-risk group. Supplementary Figure S3K presents a heatmap of the expression of these genes, while Supplementary Figure S3L highlights the differential expression of immune infiltration-related genes between the risk groups.

To further assess the value of the risk score for predicting immune checkpoint blockade (ICB) efficacy, The analysis includes PD1 and CTLA4 in the IPS analysis. However, no significant differences are observed in the average IPS values for PD1 and CTLA4 between low-risk and high-risk score groups, irrespective of their predicted response status. Finally, the high-risk group exhibits higher levels of immune dysregulation and immune exclusion reactions, providing critical insights into the immune landscape of high-risk KIRC (Supplementary Figure S4).

### Drug sensitivity analysis based on risk scores

**FIGURE 6:**
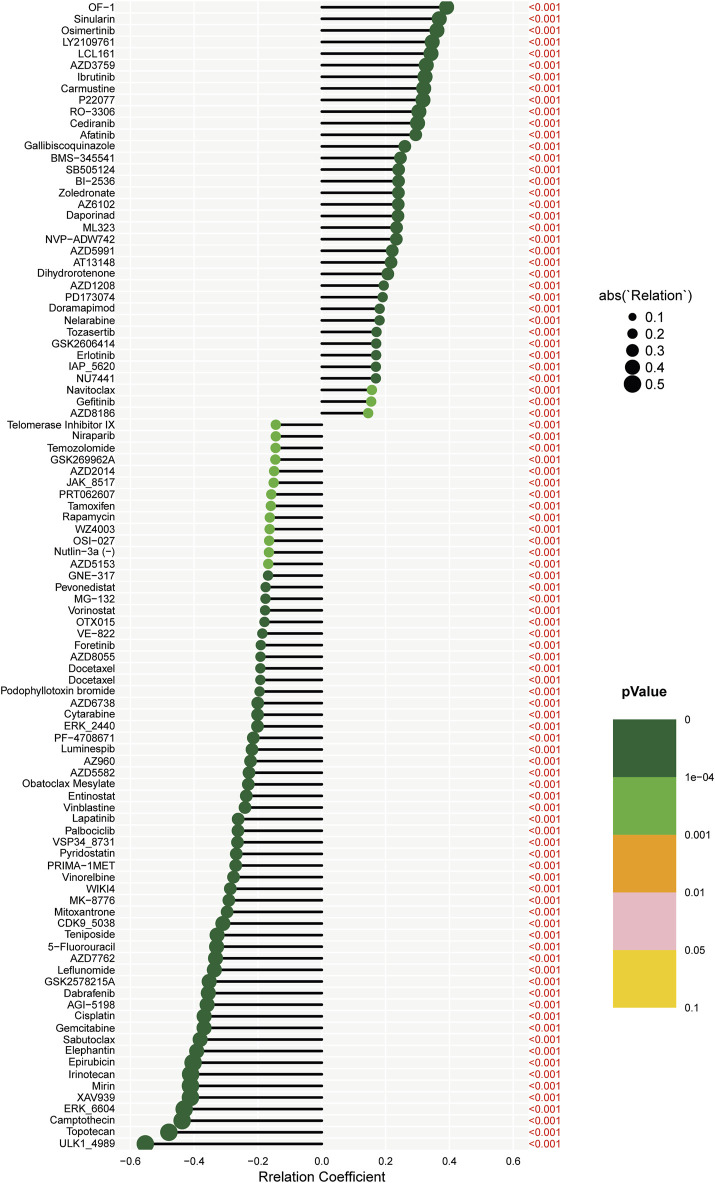
The association between risk scores and drug sensitivity within the context of KIRC.

Spearman correlation techniques were employed to examine the association between risk scores and drug sensitivity within the context of KIRC. This analysis was conducted using data from the Genomics of Drug Sensitivity in Cancer (GDSC) database. Our findings, as illustrated in Figure 6, reveal that out of a panel of drugs evaluated, 40 exhibited significant correlations with the calculated risk scores. Intriguingly, the risk score demonstrated negative correlations with 20 drugs, including ULK1_ 4989, Topotecan, and Camptothecin. This negative correlation implies that higher risk scores in KIRC patients are associated with increased sensitivity to these drugs. Conversely, the risk score showed positive correlations with the sensitivities to 20 other drugs, such as OF-1, Sinularin, and Osimertinib. This positive correlation suggests that patients with higher risk scores are less sensitive to these therapeutic agents. Among these correlative drugs, ULK1_ 4989, and OF-1 emerged as having the most pronounced impact on drug sensitivity. This finding is particularly noteworthy as it suggests that these drugs could potentially serve as key therapeutic options, with their efficacy modulated by the patient’s risk score. This information could be pivotal in guiding personalized treatment strategies for patients with KIRC, tailoring drug choices based on individual risk profiles to optimize therapeutic outcomes.

### Pathway enrichment analysis and differential pathway expression

**FIGURE 7:**
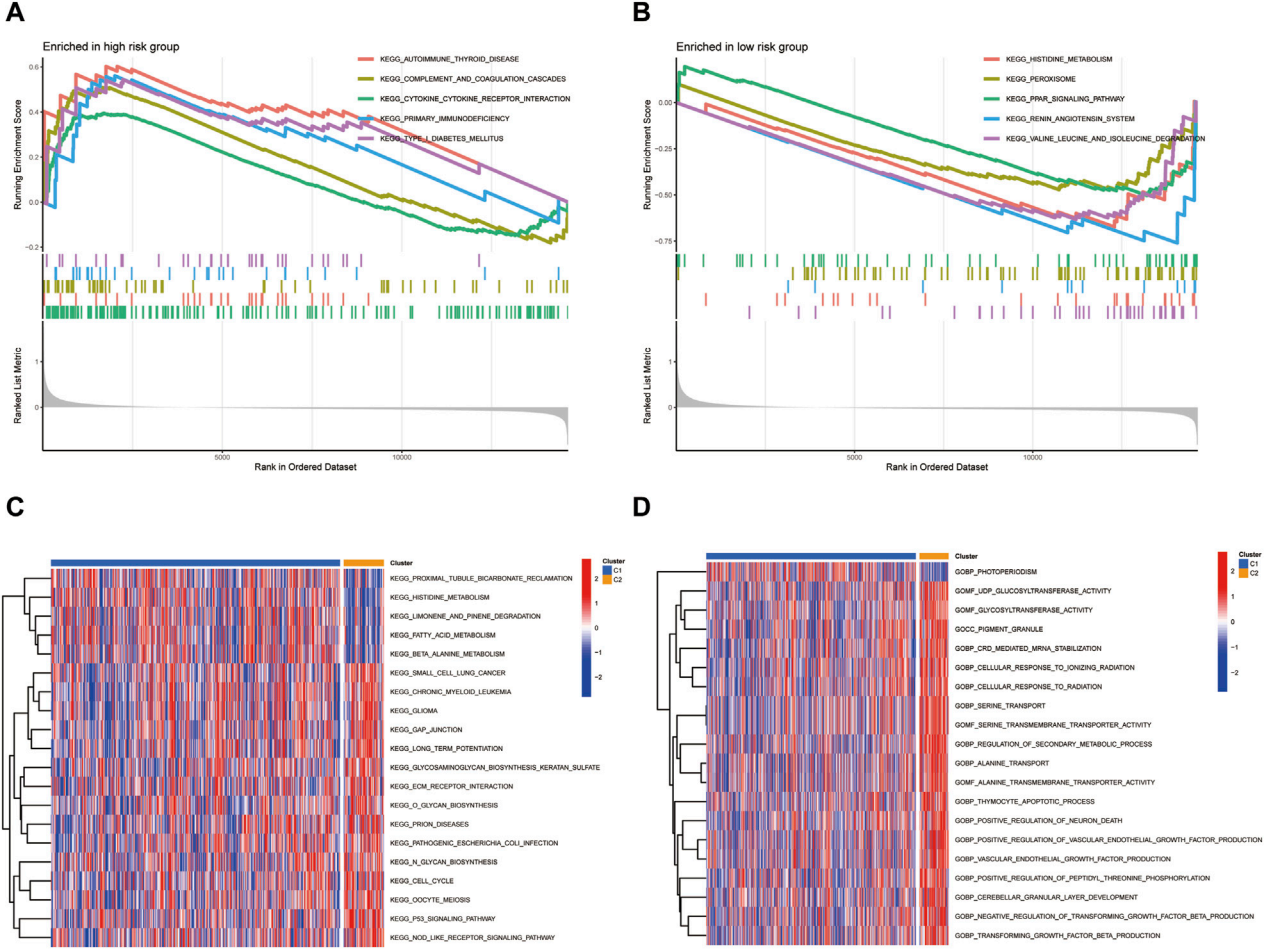
The differential pathway activation between high-risk and low-risk patient groups.**(A)** GSEA results for the high-risk group. **(B)** GSEA results for the low-risk group. **(C,D)** GSVA analysis.

We employed GSEA to delineate the differential pathway activation between high-risk and low-risk patient groups. Figure 7A elucidates the GSEA results for the high-risk group, revealing significant enrichment in several critical biological pathways. Notably, this group demonstrated pronounced enrichment in pathways associated with immune response and autoimmune disorders, such as AUTOIMMUNE THYROID DISEASE, CYTOKINE-CYTOKINE RECEPTOR INTERACTION, PRIMARY IMMUNODEFICIENCY, SYSTEMIC LUPUS ERYTHEMATOSUS, and TYPE I DIABETES MELLITUS. These findings suggest a complex interplay between KIRC progression and immune dysregulation in high-risk patients. Conversely, the low-risk group, as depicted in Figure 7B, showed distinct pathway enrichment patterns. Key pathways such as NEUROACTIVE LIGAND-RECEPTOR INTERACTION, PEROXISOME, PPAR SIGNALING PATHWAY, RENIN-ANGIOTENSIN SYSTEM, and VALINE, LEUCINE, and ISOLEUCINE DEGRADATION were notably enriched. This enrichment indicates a divergent molecular profile in low-risk KIRC patients, potentially reflecting differences in metabolic processes and hormonal interactions. Further, our GSVA presented in Figures 7C, D, highlighted additional disparities between the groups. Noteworthy differences were observed in pathways such as PROXIMAL TUBULE BICARBONATE RECLAMATION and PHOTOPERIODISM. These results underscore the heterogeneity in biological processes and pathways between high-risk and low-risk KIRC patients, offering insights that could be pivotal in tailoring patient-specific therapeutic strategies and understanding the disease’s pathophysiology.

### Differential expression of model genes between normal and KIRC cells

**FIGURE 8:**
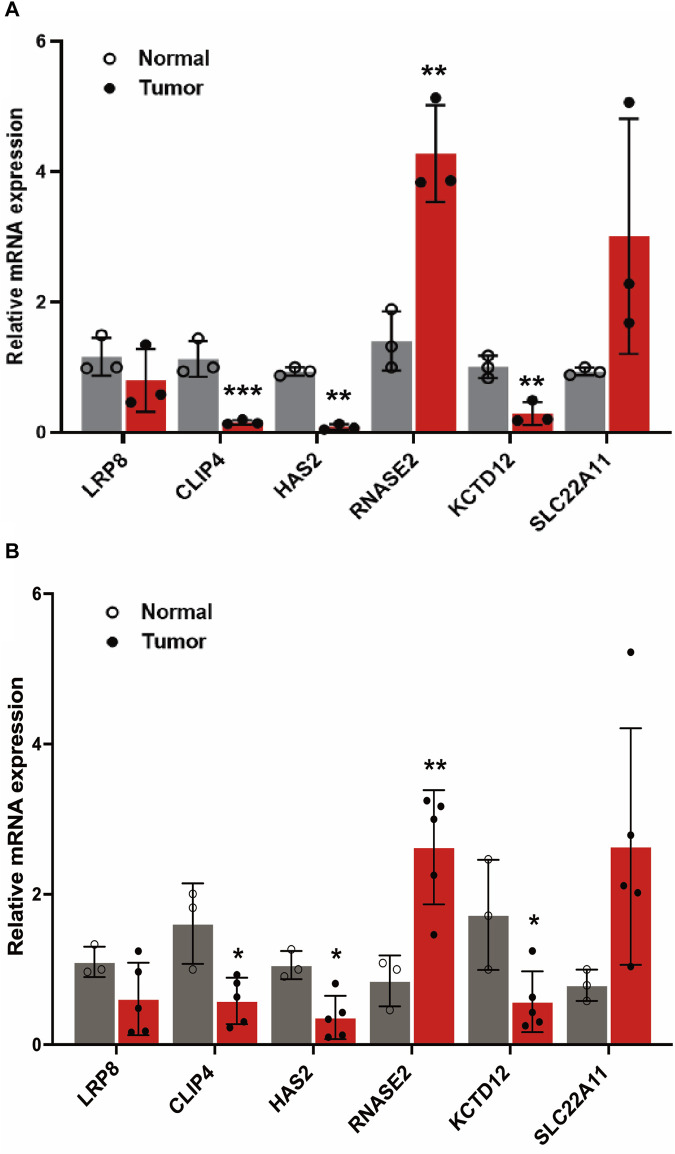
The expression level of LRP8, RNASE2, CLIP4, HAS2, SLC22A11, KCTD12 genes in cells and clinical samples. **(A)** Relative expression of 6 genes in normal human kidney cell lines (CCC-HEK-1) and KIRC kidney cancer cell lines (786-O cells) **(B)** Relative expression of 6 genes in clinical samples (*p < 0.05, **p < 0.01, and ***p < 0.001).

A comprehensive analysis was conducted to examine the differential expression of specific model genes in tumor cells and clinical samples of KIRC. The results showed that CLIP4, HAS2, RNASE2, and KCTD12 genes were statistically significant in both tumor cells and clinical samples in Figure 8. Among them, RNASE2 was significantly upregulated (*p* < 0.01, Figure 8A), and the expression results of related mRNA showed a similar trend (*p* < 0.01, Figure 8B), which suggests a potential role for these genes in the tumorigenic processes or the adaptation of the tumor environment in KIRC. The enhanced expression of these genes might be reflective of alterations in cellular processes such as metabolism, signaling, or immune response within the tumor microenvironment. Conversely, a set of genes, namely CLIP4, HAS2, and KCTD12 (*p* < 0.01), were found to be notably downregulated in tumor cells. Besides, the relevant mRNA expression in clinical samples also revealed a significant downregulation (*p* < 0.05). The decreased expression of these genes could indicate their involvement in tumor suppressive pathways or mechanisms that are disrupted in the pathogenesis of KIRC. Crucially, the differential expression patterns of these model genes were found to be consistent with the results obtained from the gene differential expression analysis based on data from TCGA database. This consistency underscores the robustness of our model to a certain extent, reinforcing the reliability of our findings. The observed expression trends of these genes in tumor versus normal cells provide valuable insights into the molecular landscape of KIRC. These findings could be instrumental in identifying potential biomarkers for diagnosis or targets for therapeutic intervention, enhancing our understanding of the disease’s molecular underpinnings.

## Discussion

KIRC is the most frequent histological subtype of renal cell carcinoma and is characterised by a relatively low prognosis and large metastatic potential. Understanding the underlying cellular and molecular mechanisms to guide biomarker discovery and intervention clinically is now a major challenge (Long et al., 2022). In recent years, as prognostic studies of KIRC have continued, the number of published articles has increased. Up to now, there have been only a few studies on disulfidptosis, which is a recently discovered new mode of cell death. Novel forms of cell death triggered by disulfide stress have important implications for cancer therapy. In contrast to common types of cell death, key genes involved in the regulation of disulfides include SLC7A11, SLC3A2, RPN1, and NCKAP1, as well as the Rac-WRC-Arp2/3 signaling pathway (Zheng et al., 2023). The investigation of the role of disulfidptosis in the prognosis of KIRC and the discovery of biomarkers is a therapeutic strategy worthy of investigation.

In this study, we performed a comprehensive analysis of disulfidptosis-related genes, tumor microenvironment, gene modules, risk stratification, immune infiltration, drug sensitivity, pathway enrichment, and differential gene expression in RCC based on the relevant literature research methods of Xu K et al. (Xu et al., 2023). Our findings reveal distinct chromosomal mappings and expression profiles of DRGs, notably the downregulation of NDUFA11 and upregulation of RPN1 in tumor tissues. The study further delves into the complexities of TME, highlighting differences in immune cell infiltrates and stemness indices between patient subgroups classified using the NMF algorithm based on the expression levels of disulfidptosis-related genes. Through hierarchical clustering and module analysis, we identified key genes and pathways associated with various biological scores and patient survival, underscoring their potential as prognostic markers. The development of a risk stratification model based on these findings provides a robust prognostic tool, as validated by Kaplan-Meier analysis, ROC curves, and Cox regression analysis using TCGA samples. Additionally, our exploration of drug sensitivities, utilizing Spearman correlation techniques, offers valuable insights for personalized treatment strategies by linking risk scores with drug responses. The study also employs GSEA to elucidate differential pathway activations between high-risk and low-risk patient groups, revealing a complex interplay between KIRC progression and immune dysregulation.

However, the study is not without limitations. The generalizability of the findings, especially regarding gene expression and drug sensitivity, may be constrained. The intricate nature of TME in KIRC warrants a more detailed analysis, and the conclusions drawn, particularly in subgroup analyses, could be limited by the cohort size. Furthermore, the correlation between risk scores and drug sensitivity does not necessarily imply causation and requires deeper investigation. Translating these findings into clinical practice faces challenges, including the need for validation in larger, more diverse cohorts and the integration of other clinical factors.

Despite these limitations, our study provides a detailed overview of the genomic and molecular landscape of KIRC, identifies potential biomarkers for prognosis and therapeutic targets, and underscores the significance of TME and immune infiltration in KIRC. What’s more, this study provides valuable insights for prognostic and therapeutic guidance by revealing the role of genes involved in the process of disulfuration in the prognosis and immunity of KIRC. However, our study is limited, and further experiments are needed to validate our findings and elucidate the underlying mechanisms. These findings lay the groundwork for developing personalized medicine approaches for KIRC treatment and enhance our understanding of its genetic underpinnings and complexity.

## Data Availability

The original contributions presented in the study are included in the article/Supplementary Material, further inquiries can be directed to the corresponding authors.
