# Supplementary material for: Development of a prognostic model based on different disulfidptosis related genes typing for kidney renal clear cell carcinoma
Source: Front Pharmacol. 2024 Mar 13;15:1343819. doi: 10.3389/fphar.2024.1343819 (PMC10976849; doi:10.3389/fphar.2024.1343819)
Supplement: Supplementary file 1 [file Table1.docx]

Table 1 The PCR primers

| Gene Name | Primer Sequence |
| --- | --- |
| LRP8 | 5’ -CCCATCCCTAATCTTCACCAAC-3’ |
|  | 3’-CTAGTGCCACGACATTCTTGAG-5’ |
| RNASE2 | 5’ -TTTACCTGGGCTCAATGGTTTG-3’ |
|  | 3’-TGCATCGCCGTTGATAATTGT-5’ |
| CLIP4 | 5’ -GTTCCAGACCCAGTAGATATGCC-3’ |
|  | 3’-CAAGTGACGTAAGCATTGCCT-5’ |
| HAS2 | 5’ -AAGAACAACTTCCACGAAAAGGG-3’ |
|  | 3’-GGCTGGGTCAAGCATAGTGT-5’ |
| SLC22A11 | 5’ -TATTAAGGGCAAACCAGACCAAG-3’ |
|  | 3’-CCAGCCCATAGTAGGAGATCAA-5’ |
| KCTD12 | 5’ -CCTAAGAGTGCAAGGCTGCT-3’ |
|  | 3’-ATGACCCAGCTCCCTACAGT-5’ |
