# Supplementary material for: Development of a prognostic model based on different disulfidptosis related genes typing for kidney renal clear cell carcinoma
Source: Front Pharmacol. 2024 Mar 13;15:1343819. doi: 10.3389/fphar.2024.1343819 (PMC10976849; doi:10.3389/fphar.2024.1343819)
Supplement: Supplementary file 2 [file DataSheet1.docx]

Supporting Information for

**Development of a Prognostic Model based on Different Disulfidptosis Related Genes Typing for Kidney Renal Clear Cell Carcinoma**

**Supporting Information Figure S1-S4**


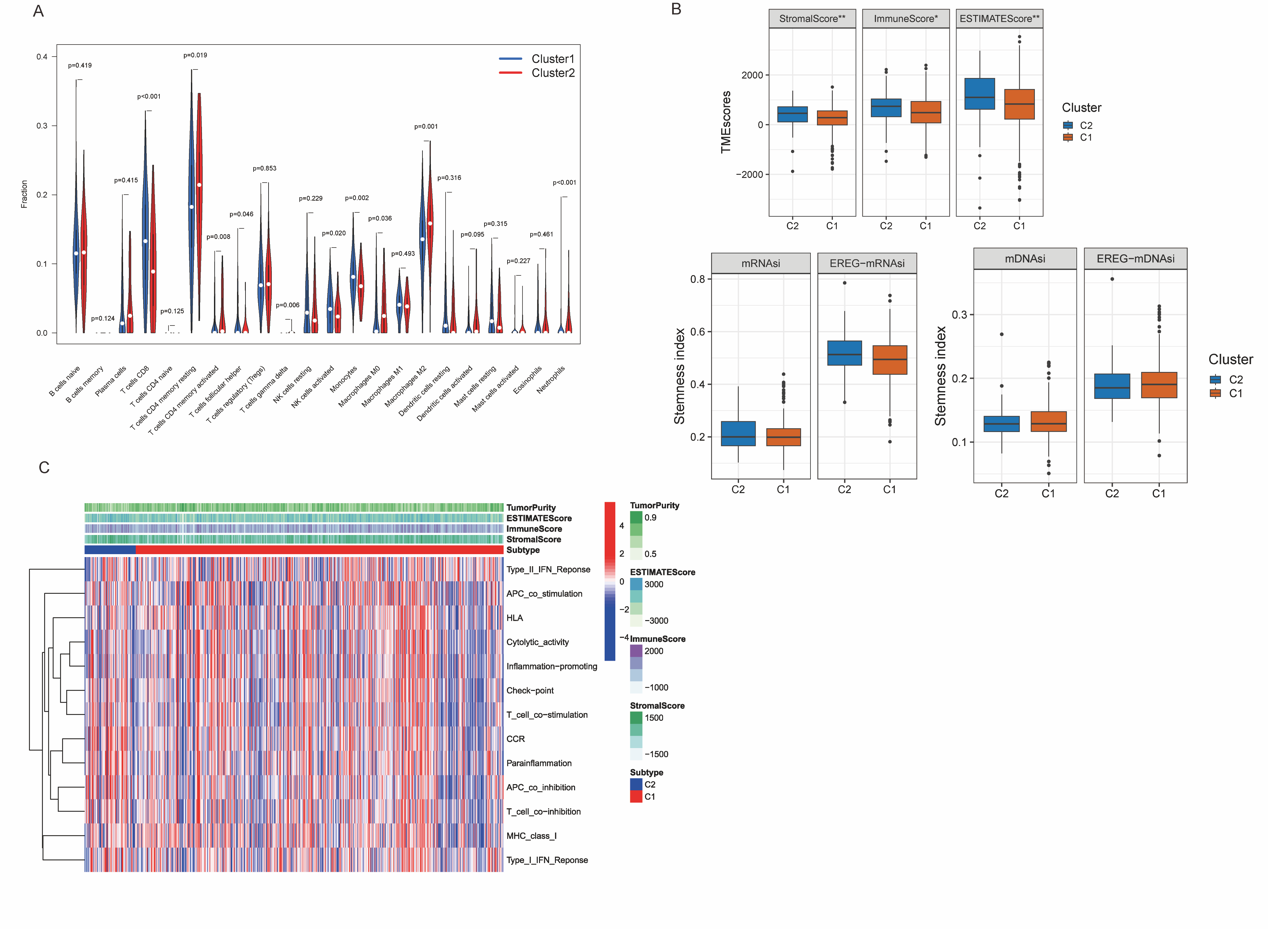


**Figure S1.** TME and stemness indices. (A) TME characterization. (B) TME scores and Stemness index. (C) The impact of clusters on immune cell infiltration in KIRC


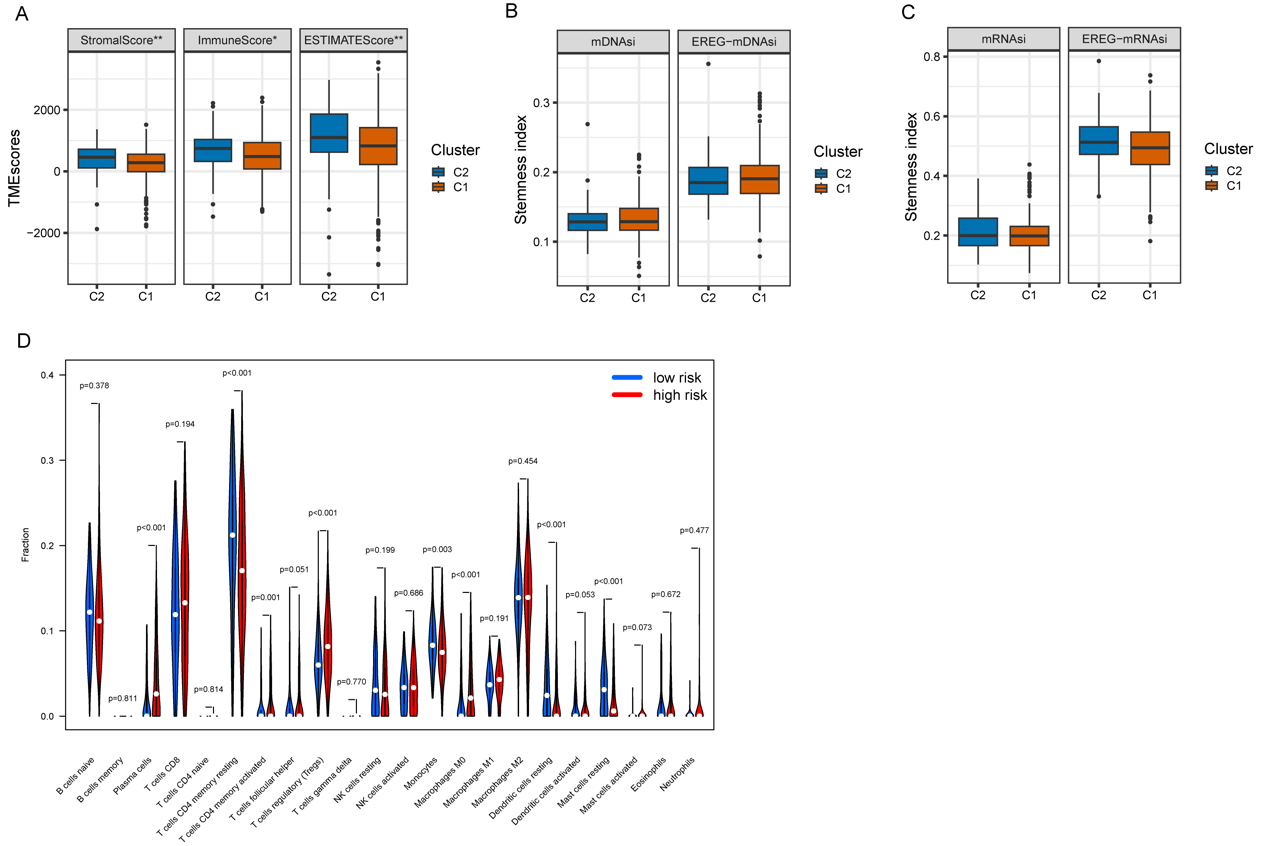


**Figure S2.** TME and genetic profiles. (A)(B)(C) TME score. (D) Strong association between the high-risk group and six types of immune infiltrating cells.


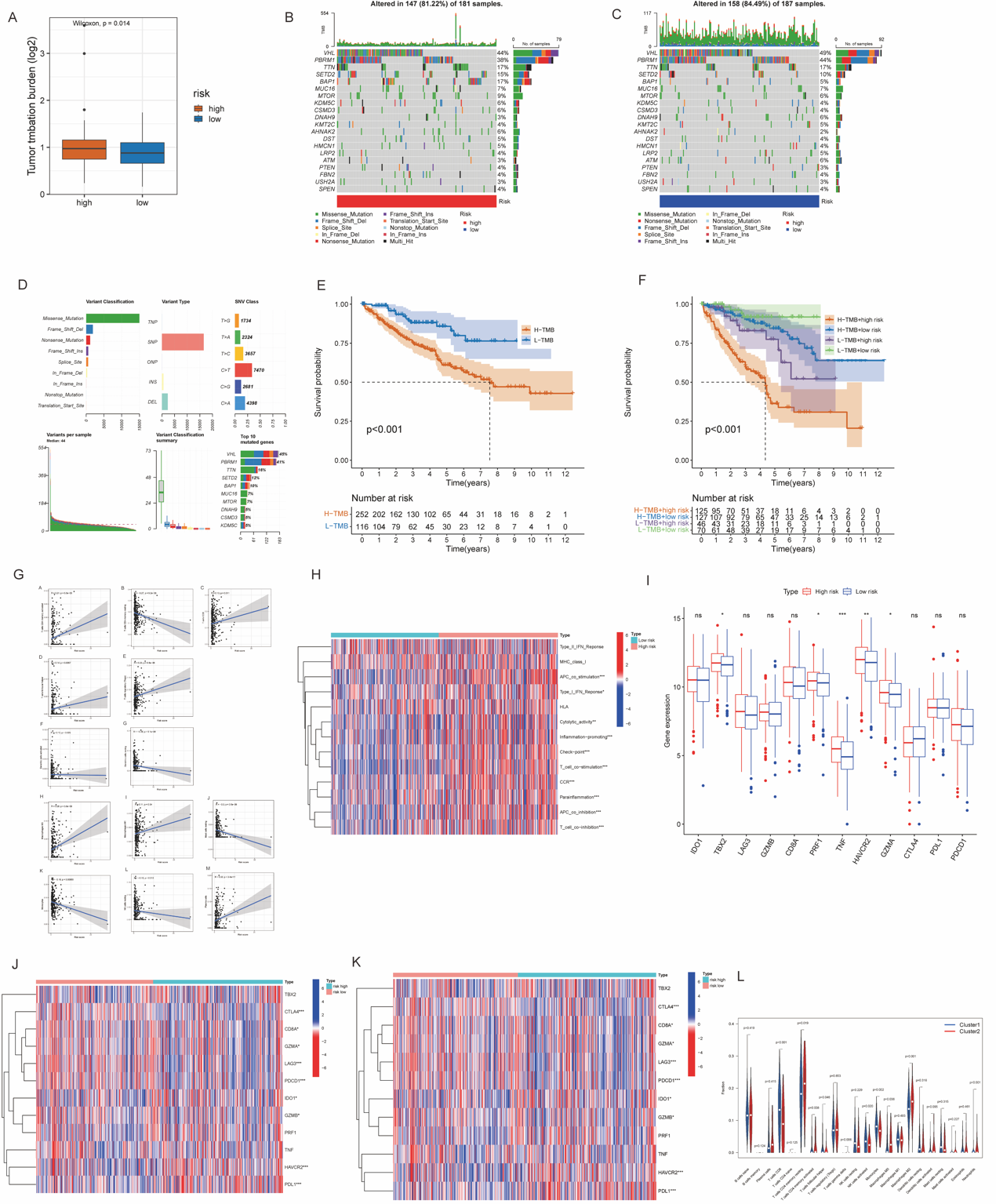


**Figure S3.** Immune Infiltration Analysis. (A) Tumor mutation burden (TMB) analysis. (B)(C) The tumor gene mutation profiles. (D) Comprehensive summary of the mutations. (E) Survival probability of H-TMB and L-TMB. (F) Comparison of survival probability in high-risk category. (G) The relationship between risk scores and immune cell infiltration. (H) (I) The differences in immune function between high-risk and low-risk groups. (J) (K) The differential expression of immune checkpoint-related genes. (L) The differential expression of immune infiltration-related genes between the risk groups.


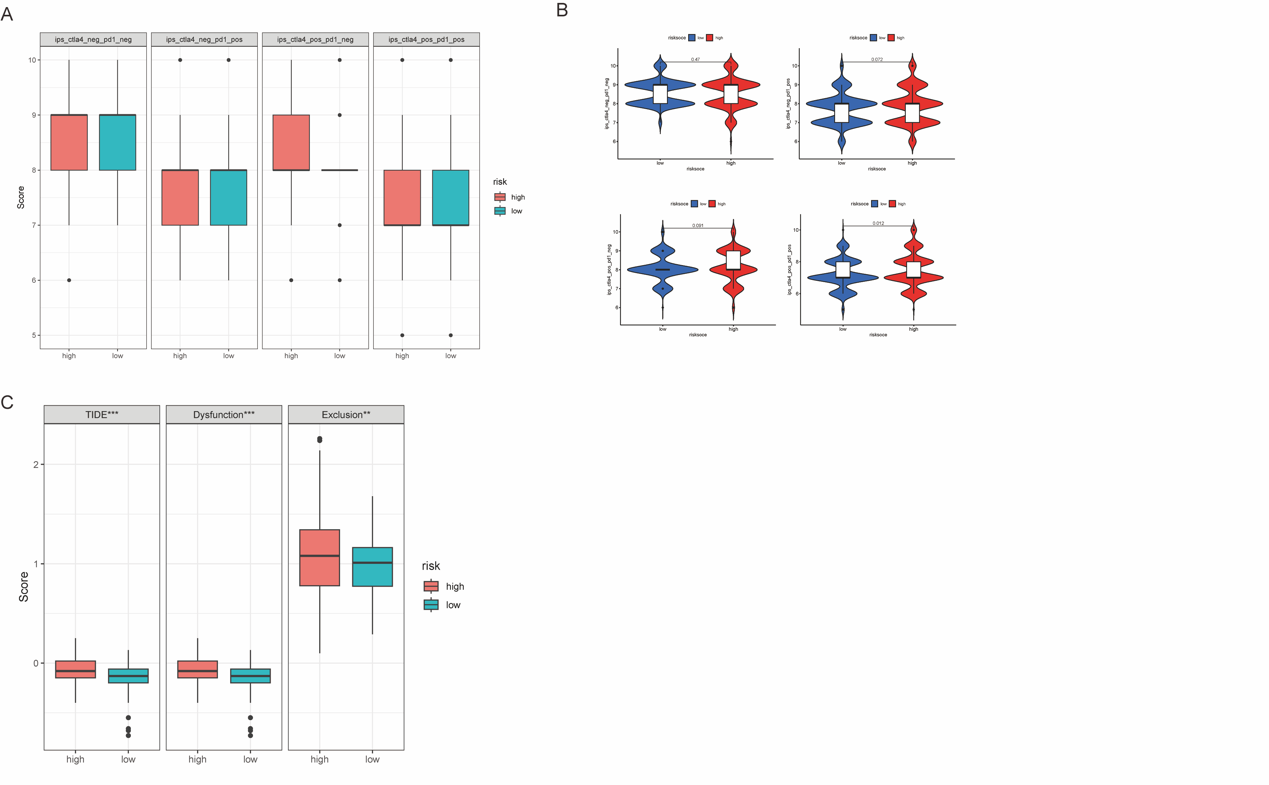


**Figure S4.** Comparative analysis of score distributions by risk levels. (A) Box plots comparing scores across three different tests, subdivided by risk categories “high” and “low”. (B) Violin plots of the score distribution's density at different risk values. (C) Box plots contrasting the score distribution between “high” and “low” risk categories.
